# Dr. Pliny P. Gordon

**Published:** 1904-04

**Authors:** 


Dr. Pliny P. Gordon, of Hobart, Ind., University of Buffalo, 1865, died March 9, 1904, aged 68 years.

